# A novel approach for geographical risk mapping of morbidity and mortality rates: the case of Val D’Agri, Italy

**DOI:** 10.1038/s41598-019-46479-z

**Published:** 2019-07-17

**Authors:** Andrea Duggento, Nicola Toschi, Antonio Pietroiusti, Loredana Musmeci, Ersilia Buonomo, Stefania Moramarco, Francesca Lucaroni, Paolo Boffetta, Leonardo Palombi

**Affiliations:** 1 Department of Biomedicine and Prevention, University of Rome Tor Vergata, Rome, Italy; 2 Hench Legal & Compliance Consulting, Rome, Italy; 3 Tisch Cancer Institute, Icahn School of Medicine at Mount Sinai, New York, NY USA

**Keywords:** Epidemiology, Risk factors

## Abstract

While associations between exposure to air pollutants and increased morbidity and mortality are well established, few rigorous studies on this issue are available. The aim of the current study is to implement a new approach to the spatial analysis of mortality and morbidity, based on testing for the presence of the same association in other areas of similar size. Additionally, we perform a case study in Val d’Agri (VA), an area of Basilicata Region, Southern Italy, where oil and natural gas extraction began in 1998. In order to examine the spatial distribution of morbidity and mortality in the region of interest, Hospital discharge (2001–2013) and mortality (2003–2014) rates for the main environment-related diseases were calculated. In addition, a comparison between the period 1980–1998 and the period 1999–2014 was performed for cardiovascular disease mortality. For the period under study, a neutral scenario emerged for cancer and respiratory diseases, where we found no differences in morbidity and mortality as compared to the national benchmark. In some cases significantly lower values (as compared to the nation-wide benchmark) were found. Conversely, a slight excess in morbidity and mortality (as compared to the nation-wide benchmark) emerged for cardiovascular diseases. Still, this excess was common to a number of municipalities in the surroundings of VA, and appeared to be already present in 1980. Higher rates of cardiovascular diseases, lower rates of neoplastic disorders no differences in mortality for respiratory causes (as compared to the nation-wide benchmark) were found in multiple areas of the region, and were therefore not specific to VA. In summary, our data do not support the hypothesis of a role of industrial activities related to oil extraction in VA in determining mortality and morbidity patterns and trends.

## Introduction

An association between short and long-term exposure to air pollution and increased morbidity and mortality in exposed humans is well established. The experimental and epidemiological evidence is strongest for cancer, cardio-respiratory diseases, diabetes and neuro-degenerative disorders^1–3^. Since industrial activities may contribute to air pollution^4^, air quality is routinely monitored in areas close to large industrial plants, and health monitoring programs are frequently implemented to evaluate the possible health effects^5,6^. In this type of investigation, the occurrence of diseases associated with exposure to air pollutants^7,8^ is assessed in a populations possibly exposed to the agent(s) of interest, usually defined as residents in a given geographic area), and compared to that found in a (usually larger) unexposed reference population. Since the exposed areas are in often smaller than the unexposed areas, random fluctuations may result in unusually higher (or lower) occurrence of one of more diseases more frequently in the former than in the latter. We hypothesize that the identification and analysis of these “peaks” represent a precious resource for a correct interpretation of the causal relationship between industrial pollution and disease in the target population.

The aim of this paper is to implement a new approach in an analysis of air pollution exposure and mortality as well as morbidity. Any increase in the occurrence of a condition potentially linked to a given hazard detected in a specific geographical context should be validated by testing for the presence of the same association in other areas of similar size: when the same higher association (as compared to e.g. a nation-wide benchmark) is detected in areas without the exposure of interest, the causal nature of the relationship between exposure and effect can be questioned^9^. At the same time, possible concomitant lower rates in the frequency of diseases potentially related to the exposure of interest may represent crucial, albeit often neglected, information for a correct assessment of the risk posed by a given pollutant. This approach would limit the bias arising from testing a high number of hypotheses by means of multiple comparisons, which may result in statistically significant differences with little or misleading information.

We also posit that the application of this method may also help to avoid the error toward positive findings very often detectable in epidemiological studies on industrial pollution. Indeed, due to observational nature of these investigations, unbiased epidemiological studies are not easy to perform in this context. In addition, exogenous factors such as the media, public opinion and political pressure (which, from different perspectives, may tend to emphasize positive finding), may play a role. We tested this approach in Val d’Agri (VA), where recent epidemiological analyses have reported an excess mortality for cardiovascular diseases and linked this excess to the presence of oil extraction^10^. In the present study, we examined the reproducibility of the reported association, by applying the geographical and epidemiological corrections described above.

## Materials and Methods

**Figure 1:**
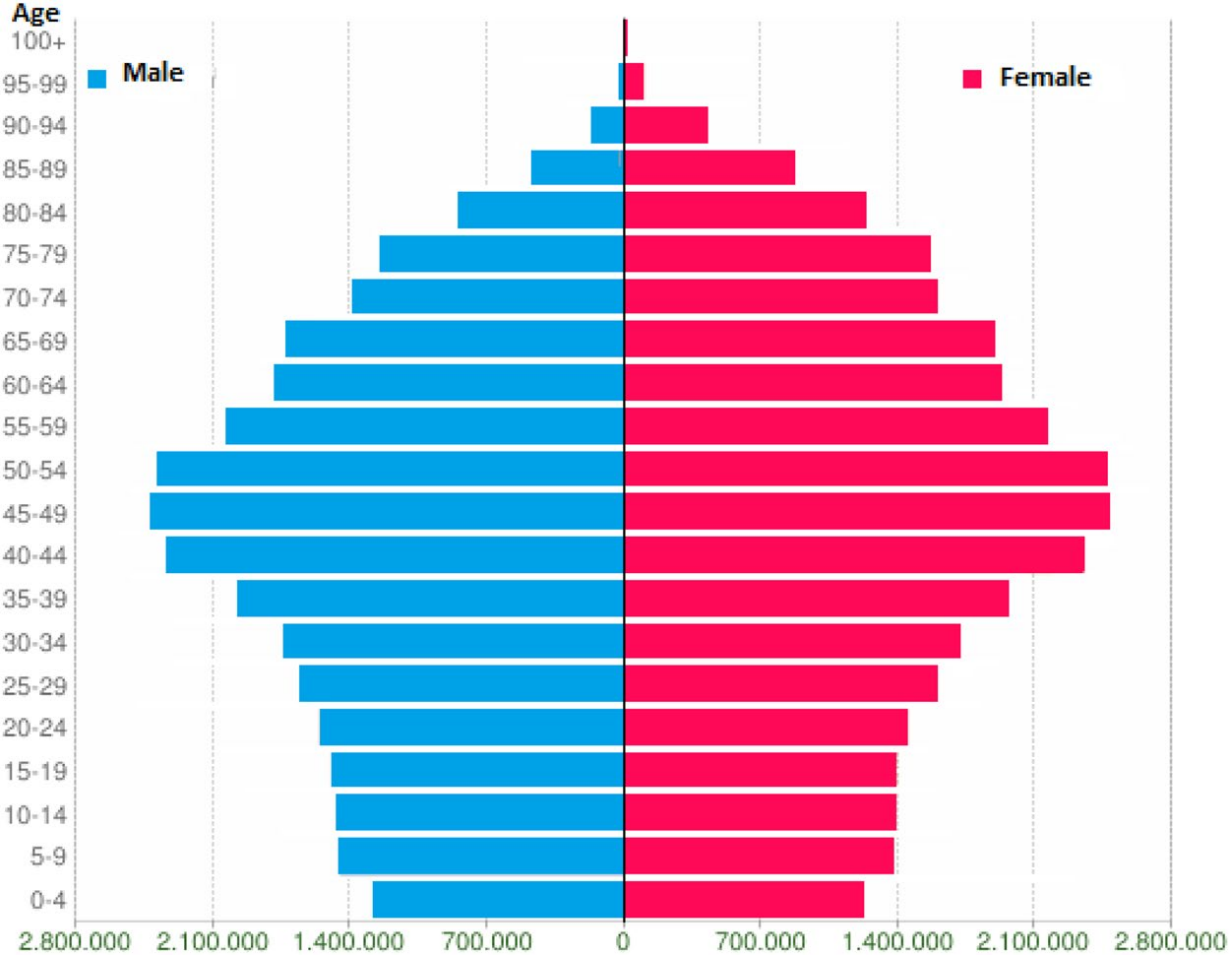
Age and sex distribution of the population of Basilicata region, 2017^11^. Left (blue bars): raw number of Italian male residents within Basilicata region; right (red bars): raw number of Italian female residents within Basilicata region.

Basilicata is a region in Southern Italy that covers about 10,000 km^2^, with a population of 570,365 in 2017^11^. Its age distribution is shown in Fig. 1. This distribution follows the typical population pyramid of high income countries, with a spinning top shape due to the progressive population ageing. Basilicata comprises two provinces and 131 municipalities (Supplementary Fig. 1); it is traditionally an agricultural region, with limited industrial infrastructure. VA comprises the municipalities of Viggiano (3,007 inhabitants in 2017) and Grumento Nova (1,701 inhabitants). In 1998, oil and natural gas extraction on industrial scale began in VA, making it the largest such operation in continental Europe, with the capacity of treating over 100,000 barrels of oil and over 4.5 million Sm^3^ of associated natural gas daily^12^. Crude oil and natural gas are separated locally from water; oil is then transferred by pipeline to a different location for refining while natural gas is cleaned locally and pumped directly into the pipeline system for delivery to the clients. No other oil extraction or refining operations are located in Basilicata region.

### Demographical standardization

In order to calculate the expected cases (hospitalizations or deaths) in the resident population, and in order to standardize the number of hospitalizations or death according to age and gender distribution in each municipality, additional demographic data was employed. Specifically, Italian Institute of Statistics (ISTAT) data regarding resident population structure on January 1 for 2012 as well as reconstructed resident population structure for other years (source: dati.istat.it). The number of residents was stratified by municipality, age range (in 10 year intervals, ages over 100 were aggregated), and gender. Other demographical indicators (e.g. marital status, nationality) were aggregated. When a low case number occurs, mortality data are censored for privacy reasons. Here we implemented an additional correction which entailed replacing censured deaths with putative causes of death sampled from the distribution of causes of death in the corresponding age and gender stratum in the Basilicata region.

### Morbidity rates

Data on hospital discharges were provided by the Italian Ministry of Health for the years 2001–2013, thus covering the time period following the beginning of the oil extraction activities in VA. We followed a previously reported approach^13^ to calculate the ratio of the hospital discharge rate for three outcomes, which have all been associated with air pollution: all malignant neoplasms (International Classification of Diseases, version 9 [ICD-9] codes 140–208), cardiovascular diseases (ICD-9 codes 390–459), and non-neoplastic respiratory diseases (ICD-9 codes 460–519) in each municipality. For every individual, only first-time first hospital discharge records were included; subsequent hospitalizations of the same patient with same ICD9 were considered a follow-up of the event that generated the first hospitalization, and therefore discarded. For each cause of hospitalization, age-standardized hospitalization rates for each municipality were computed as follows:

$${R}_{i}=\sum _{j}\frac{{h}_{ji}}{{n}_{ji}}{w}_{j}\times 100.000$$

where *R*_*i*_ is the hospitalization rate in the *i*-th municipality, *h*_*ji*_ are observed hospital discharges, *n*_*ji*_ is population in the *j*-th age and gender class, *w*_*j*_ is the proportion of standard Italian population in age class *j*. Standardized hospitalization ratios (SHR) are then defined for every municipality as the ratio between observed and expected events. For each cause of hospitalization, SHRs are calculated as:

$$SH{R}_{i}=\frac{\sum _{j}{h}_{ji}}{\sum _{j}{R}_{j}{n}_{ji}}\times 100$$

where *R*_*j*_ is the hospitalization rate in nation-wide population and in the *j*-th age class.

### Mortality rates

Cause-specific mortality rates were provided by Italian Institute of Statistics for the period 2003–2014 and recorded according to the “European shortlist of causes of death” https://ec.europa.eu/eurostat/ramon/nomenclatures/index.cfm?TargetUrl=LST_NOM_DTL&StrNom=COD_2012&StrLanguageCode=EN&IntPcKey=&StrLayoutCode=HIERARCHIC&IntCurrentPage=1). For cardiovascular disease, additional data on mortality rates since 1980 were available. For each cause of death, age-standardized mortality rates for each municipality were computed as follows:

$${T}_{i}=\sum _{j}\frac{{e}_{ji}}{{n}_{ji}}{w}_{j}\times 100.000$$

where *T*_*i*_ is the mortality rate in the *i*-th municipality, *e*_*ji*_ are observed events, *n*_*ji*_ is population in the *j*-th age and gender class, *w*_*j*_ is the proportion of standard Italian population in age class *j*. Standardized mortality ratios (SMR) are then defined for every municipality as the ratio between observed and expected events. For each cause of death, SMRs are calculated as:

$$SM{R}_{i}=\frac{\sum _{j}{e}_{ji}}{\sum _{j}{T}_{j}{n}_{ji}}\times 100$$

Where *T*_*j*_ is the mortality rate in nation-wide population and in the *j*-th age class.

### Confounding social and lifestyle factors

Since SHR and SMR in each municipality are a complex outcome which depend on lifestyle-related factors and public health service standards, these confounding factors were accounted for in our SHR and SMR estimates using a regression approach. In detail, the following confounding factors were available: Body mass index and Smoking habits (binary variables, defined at a provincial level), and total normalized municipal expenses for social services (continuous variable defined at municipality level). Total normalized municipal expenses for social services, include local health care assistance (homeless assistance, home care, meal vouchers, housing contributions), as well as social and family services (parenting, schooling, nursery schools). This figure can be considered (amongst available data) the most complete proxy of local investment in Public Health in a broad sense, which also takes into account population frailty. Other lifestyle-related factors were not included into the analysis due to the difficulty of obtaining objective data at municipality level. SHR and SMR in each municipality was transformed into a normal variable *Y* (dependent variable) and a multiple linear regression analysis was employed to model the relationship between the confounding factors (independent variables, *X*_*j*_) and dependent variable:

$$Y=K+\sum _{i}{\beta }_{i}{X}_{i}+\varepsilon $$

where *ε* is the statistical residual, *K* is the model intercept and *β*_*i*_ are regression coefficients determined numerically using the least squares method. The corrected SMR was calculated through inverse transformation from the quantity *K* − *ε* (i.e. transformed rate net of the influence of demeaned covariates).

### Statistical analysis

In order to test the null hypothesis that expected cases = observed cases (while assuming that the latter follow a Poisson Distribution), we employed the mid-p exact test as follows (see^2^):

$$\begin{array}{c}a > \lambda \,\,\,\,\,\,\,\,\,p=(\frac{1}{2})\frac{{e}^{-\lambda }{\lambda }^{a}}{a!}+\sum _{k=a+1}^{\infty }\frac{{e}^{-\lambda }{\lambda }^{k}}{k!}\\ a < \lambda \,\,\,\,\,\,\,\,\,p=(\frac{1}{2})\frac{{e}^{-\lambda }{\lambda }^{a}}{a!}+\sum _{k=0}^{a-1}\frac{{e}^{-\lambda }{\lambda }^{k}}{k!}\end{array}$$

where *a* are observed cases and λ are expected cases ($\lambda =\sum _{j}{R}_{j}{n}_{ji}$ and $\lambda =\sum _{j}{R}_{j}{n}_{ji}$ for SHR and SMR respectively). we calculated a p-value related to the. The null hypothesis is rejected when p < 0.05, in which case the standardized ratio is termed statistically significant (regardless of its value).

## Results

### All causes and cancer

**Figure 2:**
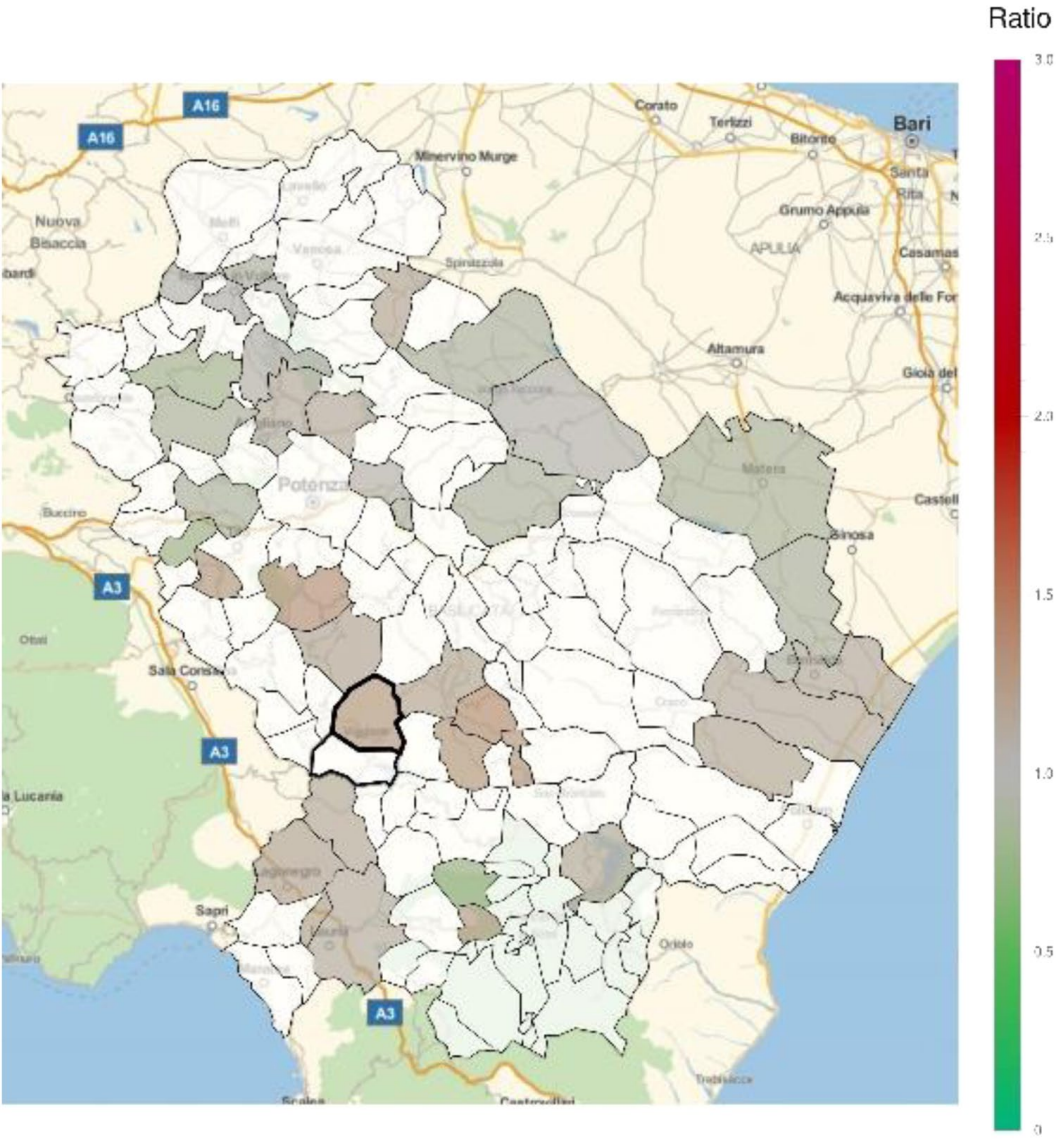
Standardized mortality ratio (SMR) for all causes of death in each municipality of Basilicata (Basilicata mortality rates with respect to nation-wide rates) for the time period 2003–2014. Green shades: SMR lower than 1, indicating a lower risk of mortality with respect to the nation-wide benchmark; Red shades: SMR larger than 1, indicating a higher risk of mortality with respect to the nation-wide benchmark. White: municipalities where SMR were not significantly different from 1. The area of Val d’Agri is highlighted by thick borders.

**Figure 3:**
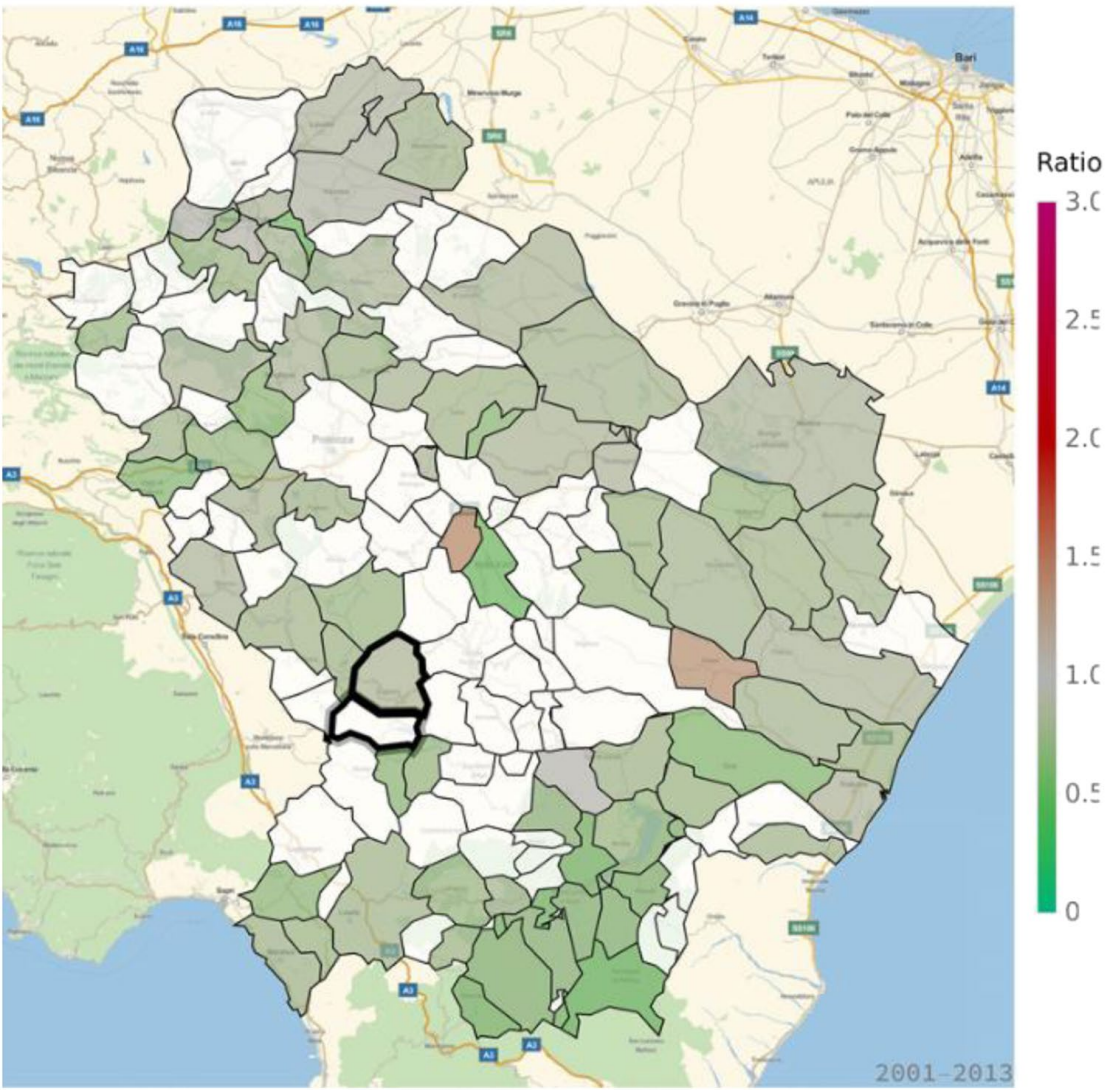
Standardized hospitalization ratio (SHR) for cancer in each municipality of Basilicata (Basilicata morbidity rates with respect to national morbidity rates) for the time period 2001–2013. Green shades: SHR lower than 1, indicating a lower risk of morbidity with respect to the nation-wide benchmark; Red shades: SHR larger than 1, indicating a higher risk of morbidity with respect to the nation-wide benchmark. White: municipalities where SMR were not significantly different from 1. The area of Val d’Agri is highlighted by thick borders.

**Figure 4:**
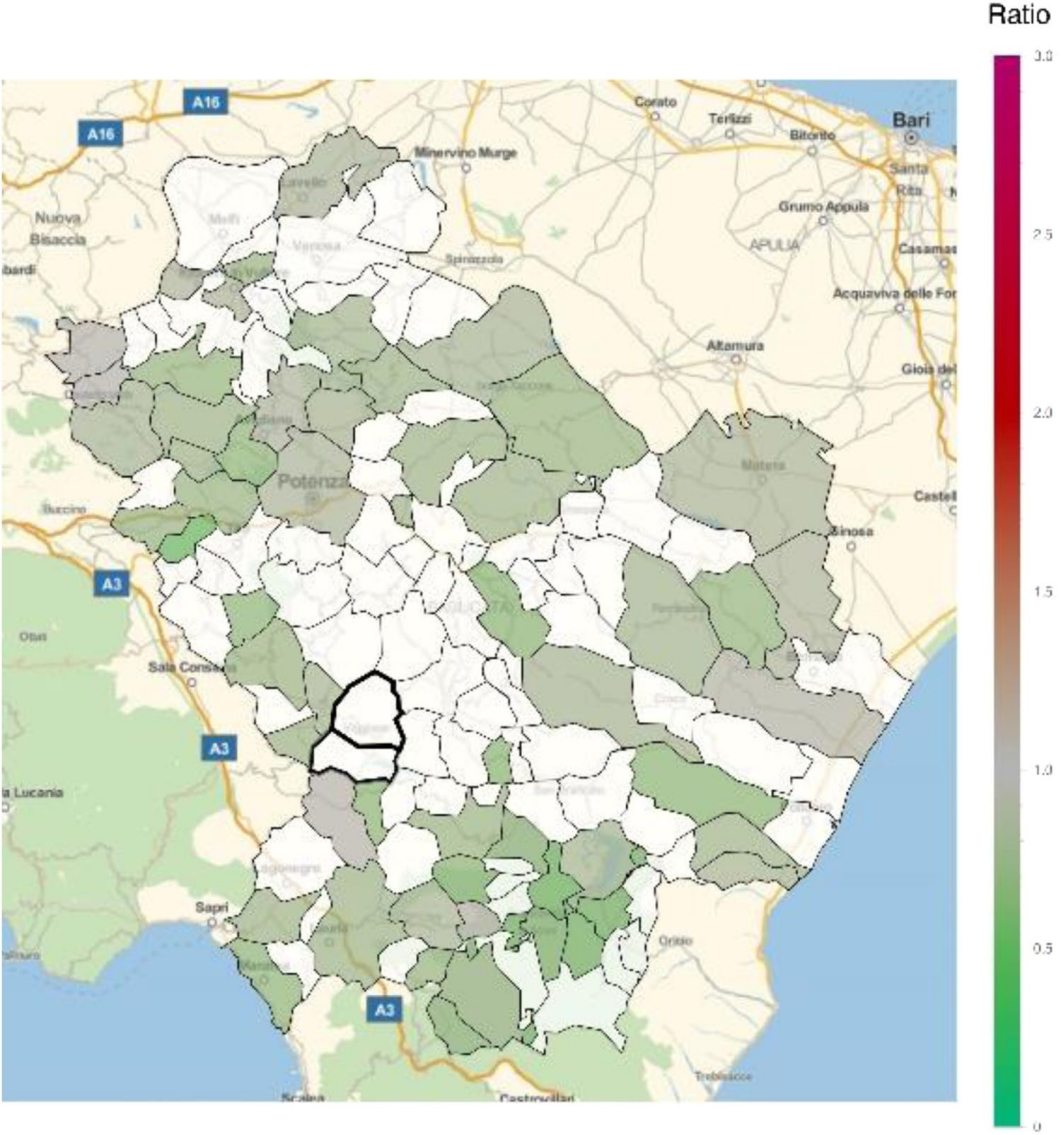
Standardized mortality ratio (SMR) for cancer in each municipality of Basilicata (Basilicata mortality rates with respect to national mortality rates) for the time period 2003–2014. Green shades: SMR lower than 1, indicating a lower risk of mortality with respect to the nation-wide benchmark; Red shades: SMR larger than 1, indicating a higher risk of mortality with respect to the nation-wide benchmark. White: municipalities where SMR were not significantly different from 1. The area of Val d’Agri is highlighted by thick borders.

The SMR for all causes were slightly higher (with respect to the nation-wide benchmark), although not statistically significant, in one of the VA municipalities compared with Italy (Fig. 2; Table 1). This higher SMR was shared by a remarkable number of municipalities in the area, ranging from 1.001 to 1.317 (Supplementary Table 1). Focusing on specific environment-related causes, there was no difference in SHR from cancer in VA compared to Italy (Fig. 3; Table 1), whereas the analysis of cancer mortality suggested a favorable ratio in VA (Table 1), like in other areas of the region (Fig. 4; Supplementary Table 2). Of note, there was no statistically significant increase in cancer mortality in any municipality of the region (Supplementary Table 2).

**Table 1:** Standardized mortality ratios (SMR) and standardized hospitalization ratios (SHR) in Viggiano and Grumento Nova for all causes and for cancer, cardiovascular diseases, and respiratory disorders.

Outcome	Measure of association	Viggiano	Grumento Nova
All causes	SMR	0.992 (*p* = 0.196)	1.091 (*p* = 0.276)
Cancer	SMR	0.750 (*p* = 0.007)	0.983 (*p* = 0.341)
	SHR	0.863 (*p* = 0.011)	0.902 (*p* = 0.163)
Cardiovascular diseases	SMR	1.192 (*p* = 0.035)	1.160 (*p* = 0.210)
	SHR	1.445 (*p* = 0.003)	1.166 (*p* = 0.017)
Respiratory disorders	SMR	1.311 (*p* = 0.102)	1.399 (*p* = 0.169)
	SHR	1.501 (*p* = 0.003)	0.907 (*p* = 0.384)

### Cardiovascular diseases

**Figure 5:**
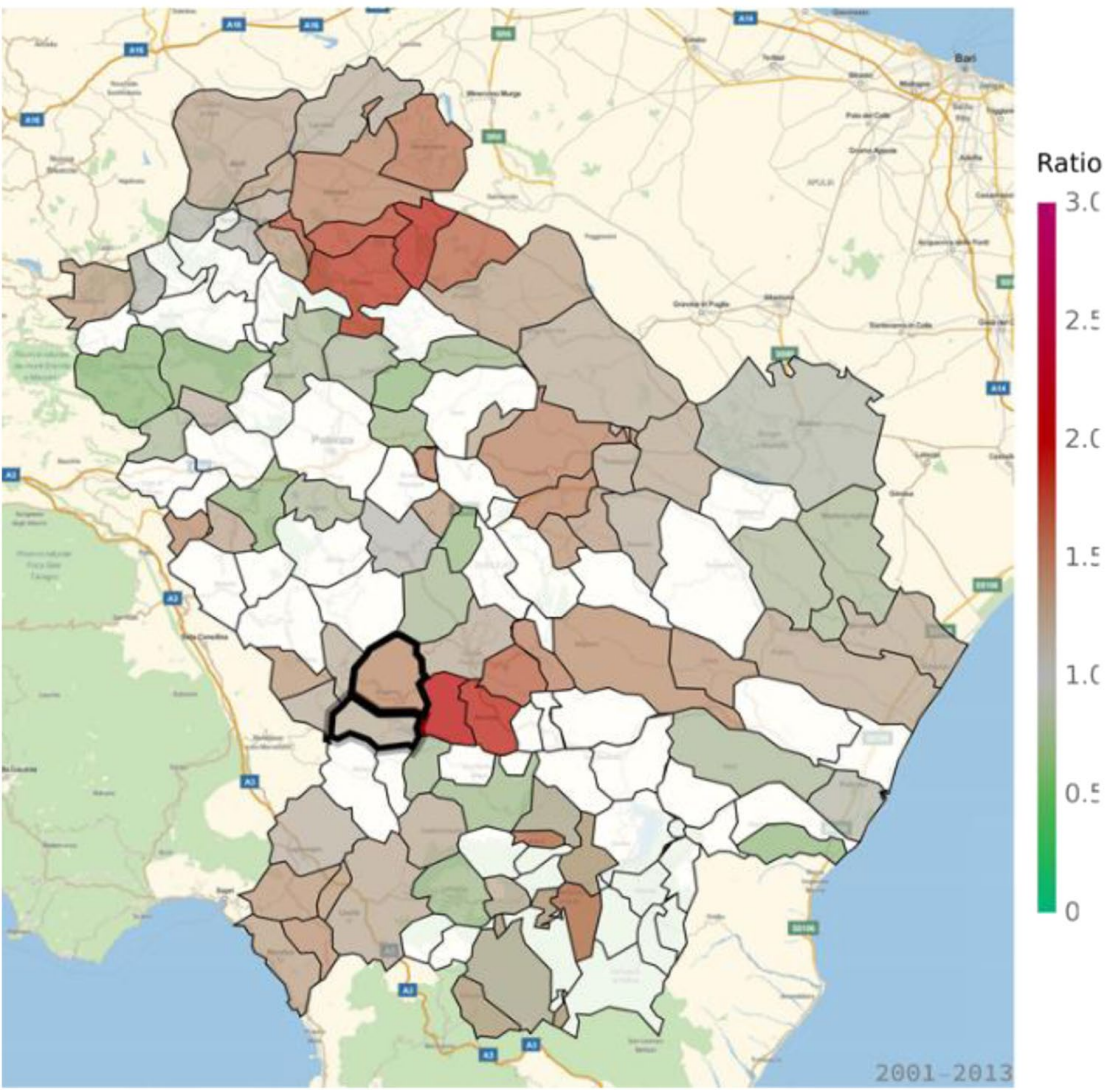
Standardized hospitalization ratio (SHR) for cardiovascular diseases in each municipality of Basilicata (Basilicata morbidity rates with respect to national morbidity rates) for the time period 2001–2013. Green shades: SHR lower than 1, indicating a lower risk of morbidity with respect to the nation-wide benchmark; Red shades: SHR larger than 1, indicating a higher risk of morbidity with respect to the nation-wide benchmark. White: municipalities where SMR were not significantly different from 1. The area of Val d’Agri is highlighted by thick borders.

**Figure 6:**
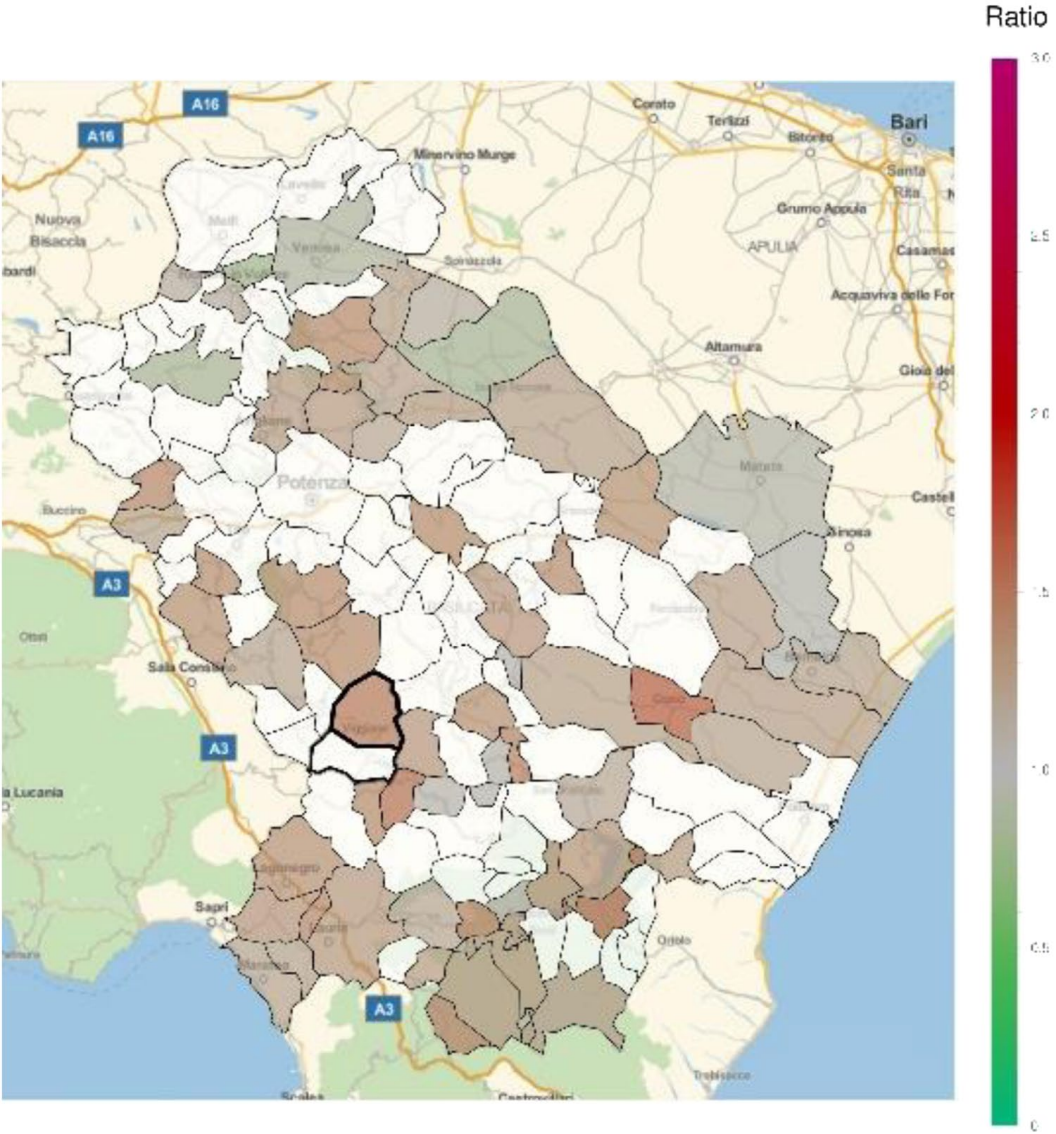
Standardized mortality ratio (SMR) for cardiovascular diseases in each municipality of Basilicata (Basilicata mortality rates with respect to national mortality rates) for the time period 2003–2014. Green shades: SMR lower than 1, indicating a lower risk of mortality with respect to the nation-wide benchmark; Red shades: SMR larger than 1, indicating a higher risk of mortality with respect to the nation-wide benchmark. White: municipalities where SMR were not significantly different from 1. The area of Val d’Agri is highlighted by thick borders.

**Figure 7:**
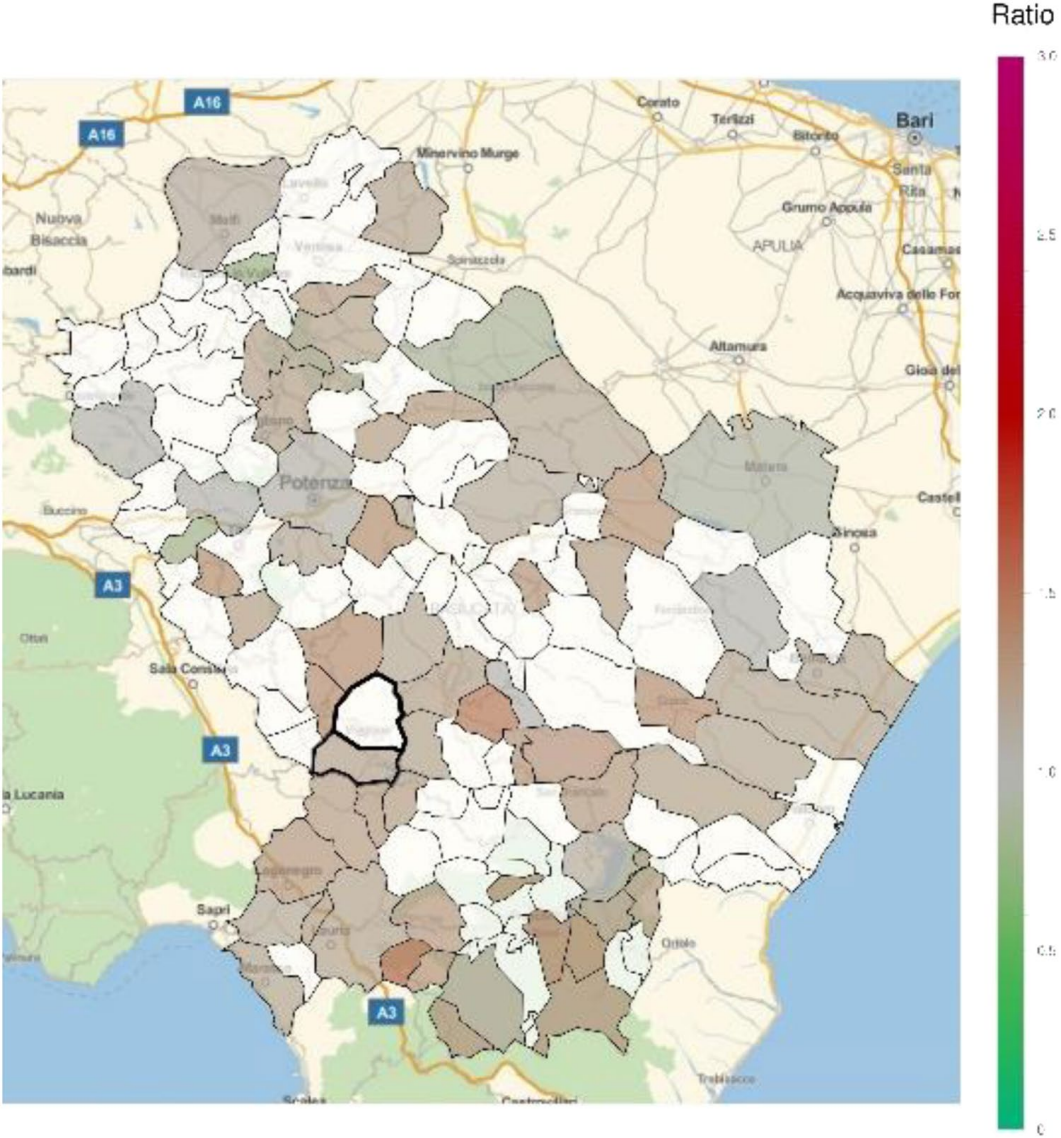
Standardized mortality ratio (SMR) for cardiovascular diseases in each municipality of Basilicata (Basilicata mortality rates with respect to national mortality rates) for the time period 1980–1999. Green shades: SMR lower than 1, indicating a lower risk of mortality with respect to the nation-wide benchmark; Red shades: SMR larger than 1, indicating a higher risk of mortality with respect to the nation-wide benchmark. White: municipalities where SMR were not significantly different from 1. The area of Val d’Agri is highlighted by thick borders.

**Figure 8:**
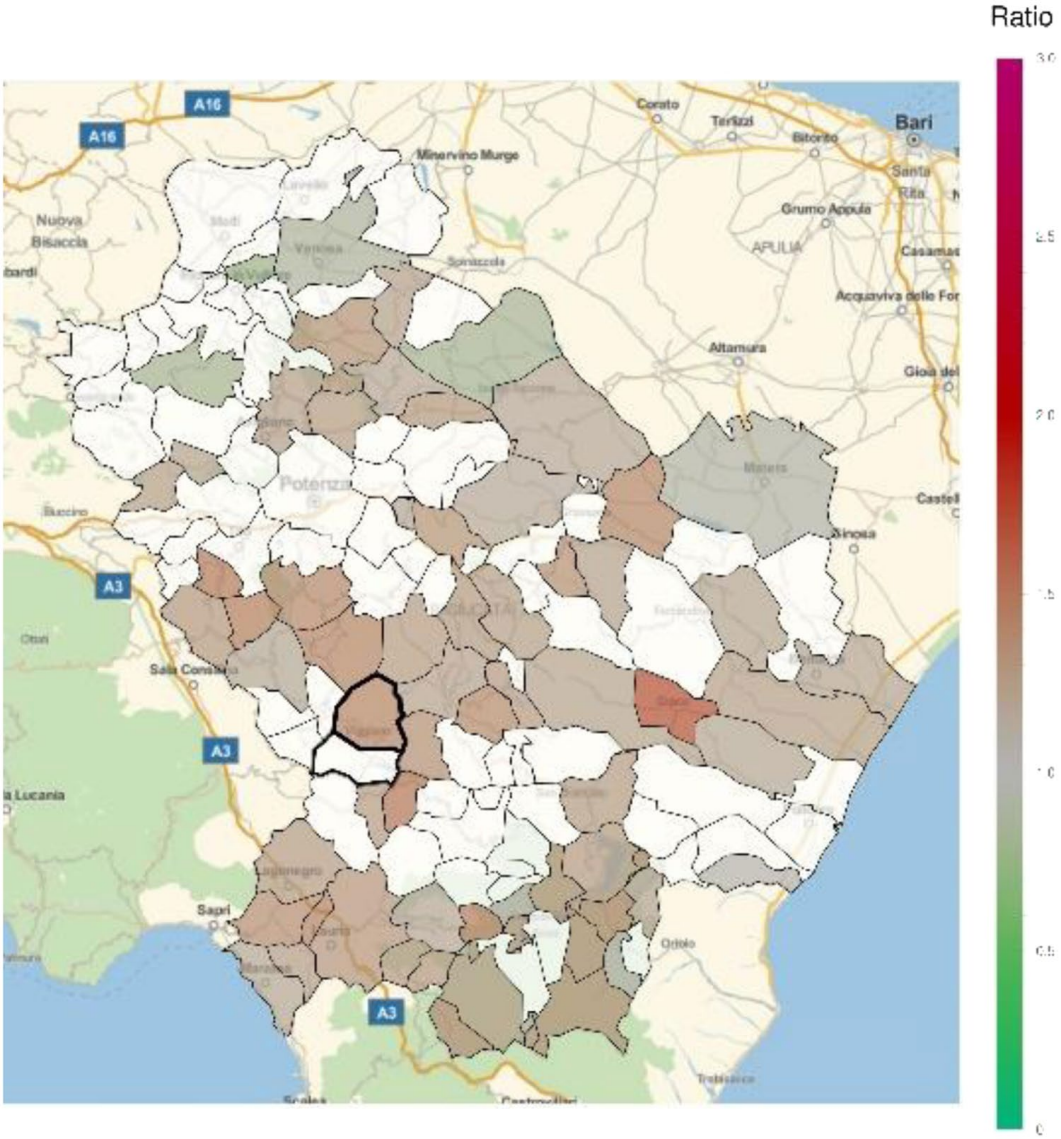
Standardized mortality ratio (SMR) for cardiovascular diseases in each municipality of Basilicata (Basilicata mortality rates with respect to national mortality rates) for the time period 1999–2014. Green shades: SMR lower than 1, indicating a lower risk of mortality with respect to the nation-wide benchmark; Red shades: SMR larger than 1, indicating a higher risk of mortality with respect to the nation-wide benchmark. White: municipalities where SMR were not significantly different from 1. The area of Val d’Agri is highlighted by thick borders.

An excess in morbidity and mortality from cardiovascular diseases was detected in VA and several other areas of Basilicata, in particular in the Northern, Eastern and Southern parts of the region (Figs 5 and 6; Table 1; Supplementary Tables 3, 4). In addition, an excess in cardiovascular disease hospitalization was present in several municipalities in the North and immediately East to VA (Supplementary Table 3). Figures 7 and 8 show the mortality rate ratio from cardiovascular diseases for the periods 1980–1998 and 1999–2014, respectively (Table 2; Supplementary Tables 5, 6). A significantly higher mortality for cardiovascular disease (as compared to the nation-wide benchmark) is evident since 1980, in particular in municipality of Grumento Nova (SMR 1.177; *p* = 0.021), as shown in Fig. 7 and Table 2. Furthermore, there is a declining trend between the two periods (data not shown), which however was not statistically significant (rate ratio 0.80; 95%CI 0.45–1.14).

**Table 2:** Comparison of standardized mortality ratios (SMR) for cardiovascular diseases in Viggiano and Grumento Nova between the period 1980–1998 and 1999–2014.

Period	Viggiano		Grumento Nova	
	SMR	*p value*	SMR	*p value*
1980–1998	0.921	0.216	**1.177**	0.021*
1999–2014	1.112	0.191	1.176	0.122

### Respiratory diseases

**Figure 9:**
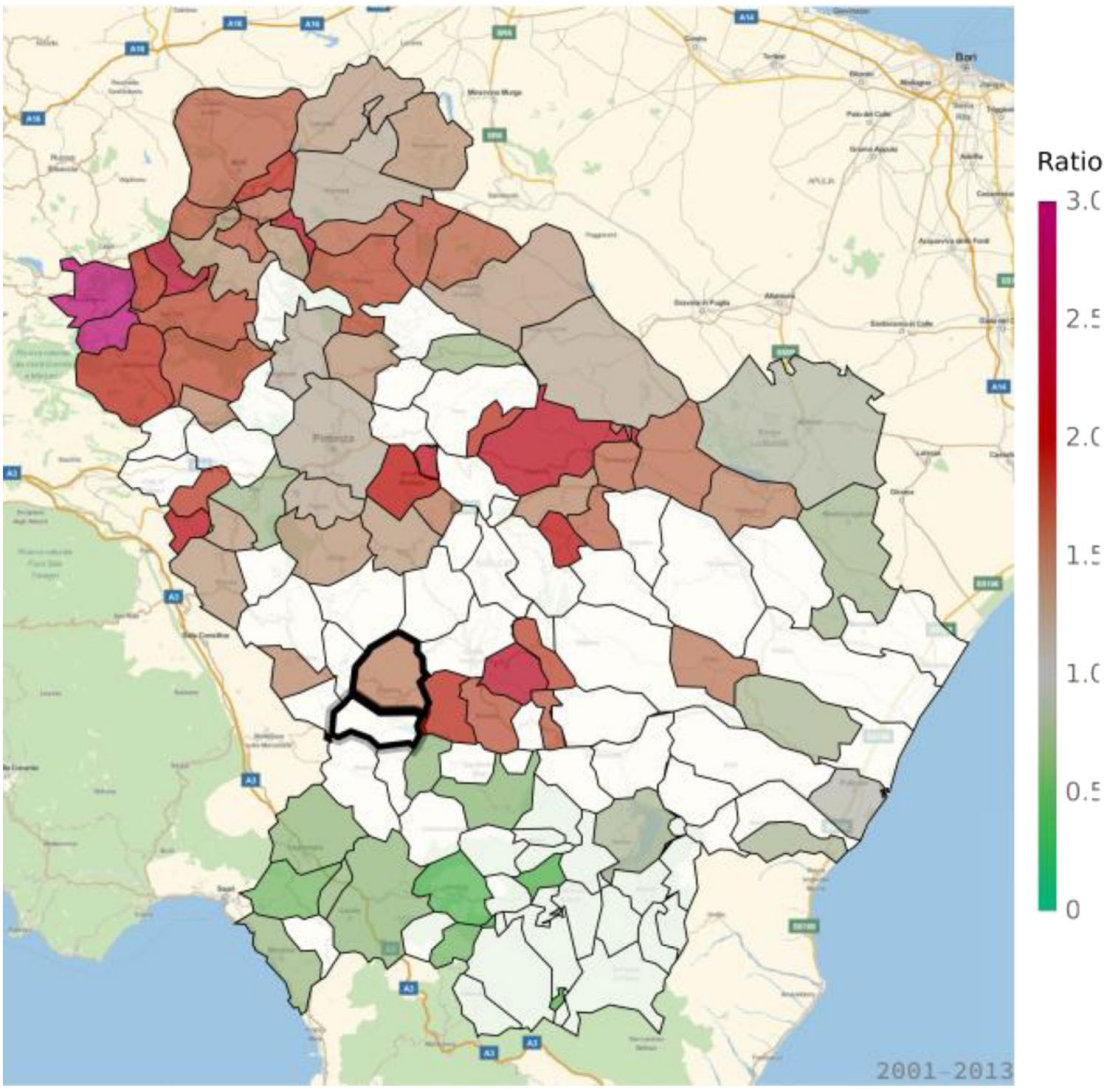
Standardized hospitalization ratio (SHR) for respiratory diseases in each municipality of Basilicata (Basilicata morbidity rates with respect to national morbidity rates) for the time period 2001–2013. Green shades: SHR lower than 1, indicating a lower risk of morbidity with respect to the nation-wide benchmark; Red shades: SHR larger than 1, indicating a higher risk of morbidity with respect to the nation-wide benchmark. White: municipalities where SMR were not significantly different from 1. The area of Val d’Agri is highlighted by thick borders.

**Figure 10:**
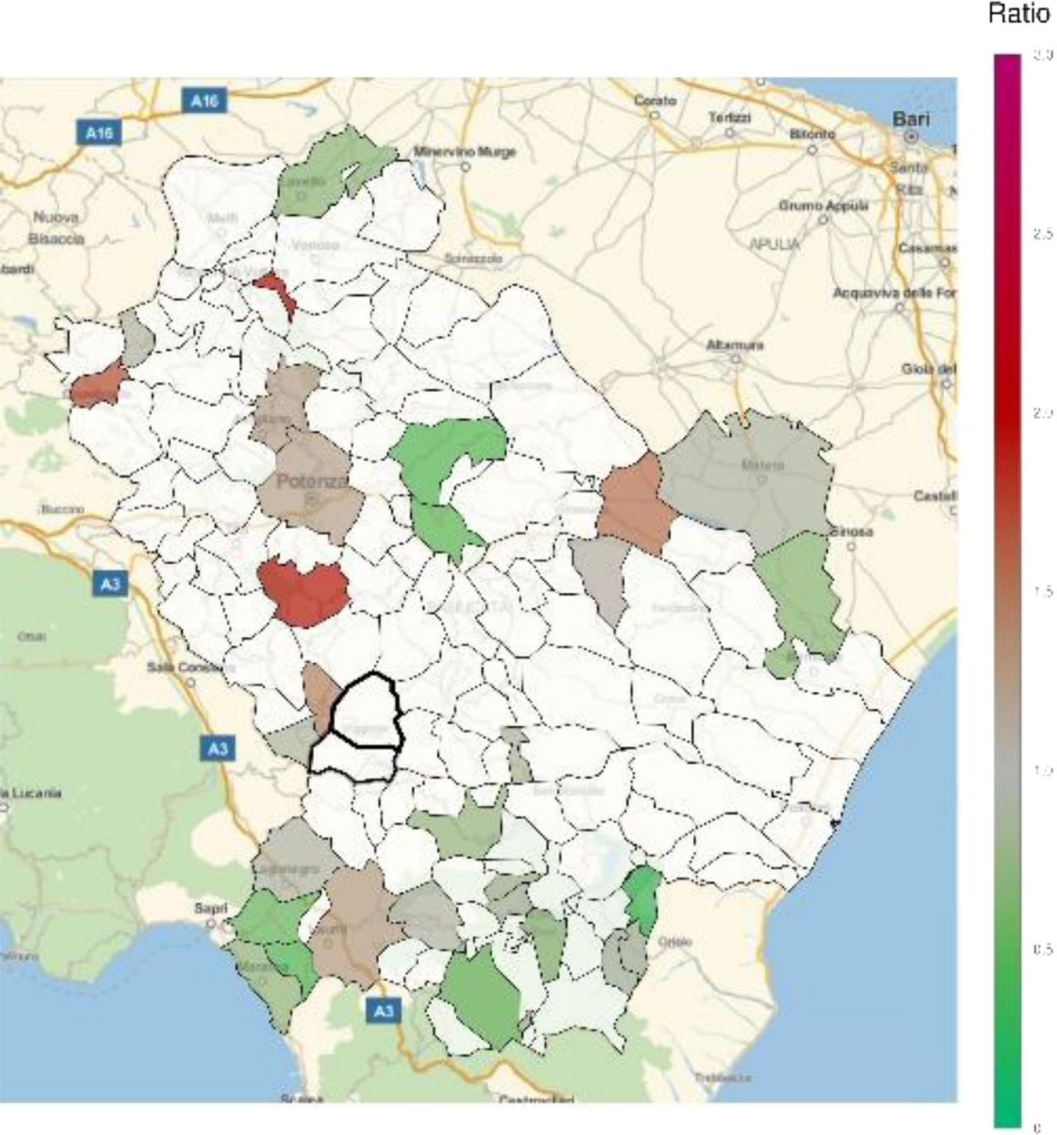
Standardized mortality ratio (SMR) for respiratory diseases in each municipality of Basilicata (Basilicata mortality rates with respect to national mortality rates) for the time period 2003–2014. Green shades: SMR lower than 1, indicating a lower risk of mortality with respect to the nation-wide benchmark; Red shades: SMR larger than 1, indicating a higher risk of mortality with respect to the nation-wide benchmark. White: municipalities where SMR were not significantly different from 1. The area of Val d’Agri is highlighted by thick borders.

The analysis of respiratory disease morbidity (Fig. 9; Table 1) showed higher morbidity (as compared to the nation-wide benchmark) in various areas of the region, in particular in its Northern part of Basilicata region, with SHR for hospitalization exceeding 3 (Supplementary Table 7). No difference in mortality for respiratory diseases with respect to the nation-wide benchmark was detected in VA and in the surrounding municipalities (Fig. 10; Supplementary Table 8).

## Discussion

The main result of our analysis was that VA did not show a specific pattern in disease burden compared to other municipalities of Basilicata region. Small excesses in general mortality rate, respiratory morbidity and cardiovascular morbidity and mortality (the latter was present since 1980) are shared with many other areas of the region, and the other indicators we analyzed are in general more favorable in VA than in most other areas of the region. From this point of view, our analysis provides no evidence in support of the hypothesis that the oil extraction operation in VA has had an impact on the burden of the disease we included in our analysis, which represent the main indicators of long-term health effects of air pollution.

In general, most areas of Basilicata revealed a favorable pattern of morbidity and mortality compared to Italy. In particular, the low morbidity and mortality from neoplasms is in keeping with previous reports^14,15^ and most likely explained by historical characteristics of behavioral and lifestyle factors, including low smoking rates among women^16^, high adherence to Mediterranean diet^17^, and low prevalence of overweight and obesity^18^. This favorable pattern is changing, as it is shown by the comparison of cardiovascular mortality ratios in the periods 1980–1998 and 1999–2014 (Figs 7 and 8), which shows a trend toward a decreasing number of municipalities with lower mortality and an increasing number of municipalities with higher mortality. This trend, which is common with other areas of Southern Italy and in general Southern European countries^19^, can be explained by the abandonment of a traditional lifestyle in favor of a “westernized” approach^17^, and does not seem to be linked to air pollution or other factors specific to VA. Indeed, while prevalence of overweight and obesity in Northern Italy has started to decline in 2009, thanks to increasing awareness towards poor nutrition and other lifestyle risk factors, such a trend was not seen in Southern Italy, at least until 2013. Result on respiratory disease mortality are not fully consistent with those on morbidity. The absence of significant differences in mortality, compared to the national benchmark, combined with the evidence of areas with higher morbidity, mainly located in the Northern part of region, may be explained by additional exogenous factors like e.g. different distribution of hospital facilities.

Our study suffers from several limitations. First, it consists of a series of ecologic comparisons of rates, which did not take into account potential confounders such as diet and other nutritional factors, sources of indoor and outdoor air pollution, and a direct indicator of socioeconomic status, which may have contributed to the patterns identified in the analysis. In addition, several municipalities under study had less than 1,000 inhabitants, resulting in unstable rates despite the aggregation of data from more than ten years. Despite these limitations, our analysis identified several patterns that would be worth exploring more in detail, in particular for cardiovascular and non-neoplastic respiratory diseases. Our study also stresses the need to compare the results in a given area against the local background of epidemiological and geographical scenarios. The integration of life-style data and accurate information on the geographical distribution of industrial pollutants from oil extraction operations and other sources of air pollution such as road traffic, by means of appropriate diffusion models, will lead to a further refinement of our results and contribute to explain them. Our epidemiological approach can provide a contextual framework for the data obtained from a single area, such as VA. The analysis of additional variables measured in the different areas (lifestyle factors, industrial activities, geographical characteristics, as well as genetic background) would contribute to identifying and /or further refining health risk factors.

Also, while our study did not provide evidence for a health effects of industrial emissions in VA, it is plausible to hypothesize that some (possibly unmeasurable) effects related to air pollution may develop in the future in VA. However, it is important to assess which benefits may derive from further reductions in pollutant emissions compared to modifications in lifestyle factors, such as nutrition, tobacco smoking, and physical activity^20^, and which would be the costs of the different approaches. One should reason in terms of the magnitude of air pollution-related effects and the associated cost benefit, especially in comparison with other more promising health promotion interventions, especially in absence of direct evidence of an effect of air pollution on health. As mentioned above, some authors observed the abandonment of Mediterranean diet in Basilicata^17^ in the last decades, which might have been associated with the increase of cardiovascular mortality in that region.

In conclusion, sustainable development is one of the main challenges of industrialized countries. Encouraging efforts towards technological improvements associated with the lowest possible impact on human health and the environment is mandatory, however it is also important to implement state-of-the-art health promotion and prevention policies.

## Supplementary information

supplementary figure and tables
