# Supplementary material for: A novel approach for geographical risk mapping of morbidity and mortality rates: the case of Val D’Agri, Italy
Source: Sci Rep. 2019 Jul 17;9:10348. doi: 10.1038/s41598-019-46479-z (PMC6637145; doi:10.1038/s41598-019-46479-z)
Supplement: Supplementary file 1 — supplementary figure and tables [file 41598_2019_46479_MOESM1_ESM.pdf]

A novel approach for geographical risk mapping of morbidity and mortality rates: the case of Val D'Agri, Italy

Andrea Duggento (1), Nicola Toschi (1), Antonio Pietroiusti (1), Loredana Musmeci (2) , Ersilia Buonomo (1) , Stefania Moramarco (1), Francesca Lucaroni (1), Paolo Boffetta (3), Leonardo Palombi (1).

1. Department of Biomedicine and Prevention, University of Rome Tor Vergata, Rome, Italy.
2. Hench Legal & Compliance Consulting, Rome, Italy
3. Tisch Cancer Institute, Icahn School of Medicine at Mount Sinai, New York, NY, USA

*Corresponding author:
Paolo Boffetta, MD, MPH
Tisch Cancer Institute
Icahn School of Medicine at Mount Sinai
One Gustave L. Levy Place, Box 1130
New York NY 10029 USA
Tel. +1-212-824-7378
Email paolo.boffetta@mssm.edu
ORCID 0000-0002-3811-2791

Supplementary Figure 1. List of municipalities in Basilicata Region.

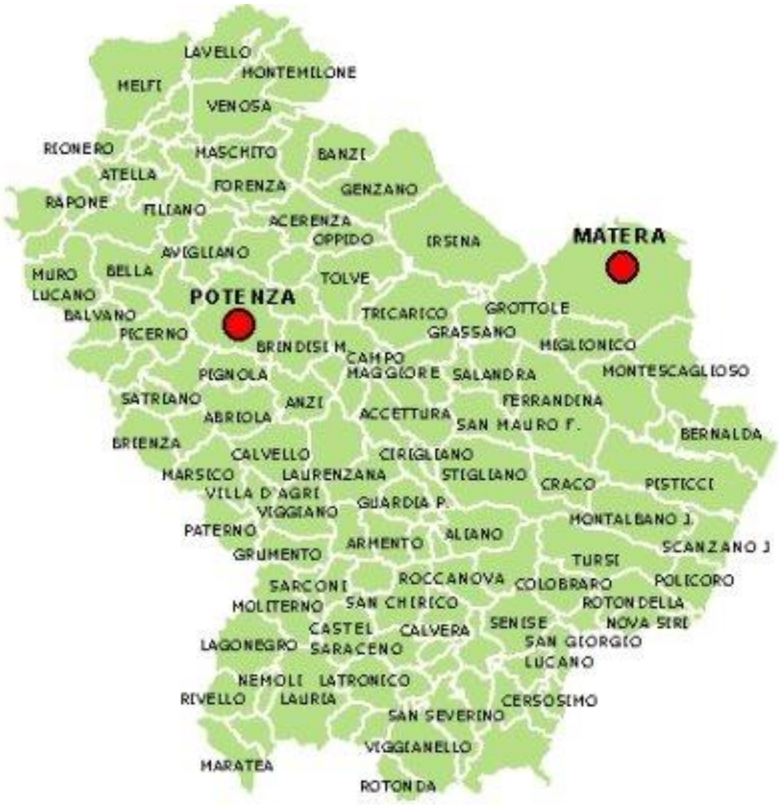

Supplementary Table 1. SMR for all causes

Municipality	SMR (vs Ita	p-value
Abriola	1.284195	0.000422
Acerenza	1.019796	0.225199
AlbanoDiLu	1.089354	0.198869
Anzi	1.04702	0.08835
Armento	1.236111	0.006131
Atella	0.942631	0.142943
Avigliano	1.085331	0.01084
Balvano	1.072651	0.355496
Banzi	0.937223	0.276089
Baragiano	0.951498	0.30205
Barile	0.939562	0.04968
Bella	0.907869	0.011962
Brienza	1.09081	0.063503
BrindisiMor	1.031704	0.262785
Calvello	1.116805	7.77E-06
Calvera	1.131172	0.185657
Campomag	1.086131	0.334274
Cancellara	1.048297	0.317939
Carbone	0.772659	0.002578
SanPaoloA	0.980002	0.249556
Castelgran	0.992407	0.286957
Castellucci	0.983469	0.1939
Castellucci	0.985158	0.110153
Castelmez	0.904434	0.102044
Castelsara	0.922718	0.206392
Castronuov	1.066589	0.417267
Cersosimo	1.011893	0.19404
Chiaromon	1.129738	0.009323
CorletoPer	1.217467	0.003996
Episcopia	0.855916	0.139253
Fardella	1.023194	0.045427
Filiano	1.026401	0.122481
Forenza	0.961459	0.181991
Francavilla	0.938319	0.493398
Gallicchio	0.922278	0.008998
GenzanoDi	1.026957	0.000213
GrumentoN	1.090808	0.276028
GuardiaPe	1.317144	0.002804
Lagonegro	1.132234	0.00277
Latronico	0.986061	0.368186
Laurenzana	1.051959	0.393489
Lauria	1.07902	0.036646
Lavello	0.971366	0.112488
Maratea	0.950615	0.138093
MarsicoNu	0.980962	0.476118
Marsicovet	1.032567	0.130887
Maschito	0.970301	0.410832
Melfi	1.028136	0.21349
Missanello	1.258761	0.016768
Moliterno	1.100118	0.037913

Montemilor	1.050661	0.40436
Montemurr	1.05296	0.384817
MuroLucar	0.94293	0.067086
Nemoli	1.168089	0.018579
Noepoli	1.054285	0.413654
OppidoLuc	1.056502	0.178183
PalazzoSa	1.123574	0.014963
Pescopaga	1.076232	0.197944
Picerno	1.052767	0.404982
Pietragalla	0.928863	0.040272
Pietraperto	1.091216	0.03349
Pignola	0.964825	0.445054
Potenza	1.04863	0.35552
Rapolla	1.000663	0.173196
Rapone	1.03959	0.278066
RioneroInV	0.98552	0.298989
Ripacandid	0.994405	0.039574
Rivello	1.044775	0.253635
Roccanova	0.995073	0.308772
Rotonda	0.878708	0.050445
Ruoti	1.035732	0.396205
RuvoDelMo	0.935736	0.18709
SanChirico	0.981394	0.390804
SanChirico	0.957065	0.476269
SanCostan	0.872718	0.061914
SanFele	0.968944	0.310661
SanMartinc	0.84448	0.004554
SanSeverir	1.016687	0.13865
SantAngelc	0.95731	0.35911
SantArcang	1.088716	0.289963
Sarconi	1.073051	0.374763
SassoDiCa	1.047292	0.230694
SatrianoDil	1.140596	0.070681
SavoiaDiLu	1.042216	0.289322
Senise	1.240866	0.000278
Spinoso	0.813195	0.009637
Teana	1.106296	0.024178
TerranovaD	1.123042	0.077796
Tito	1.116159	0.248112
Tolve	1.027858	0.150198
Tramutola	1.001743	0.114278
Trecchina	0.977031	0.454174
Trivigno	1.056728	0.419905
VaglioBasil	0.956358	0.431337
Venosa	0.984215	0.136233
VietriDiPot	1.009509	0.000374
Viggianello	0.976098	0.092184
Viggiano	0.991545	0.195698
Ginestra	1.008324	0.152275
Paterno	1.239345	0.000344
Accettura	1.034905	0.220651
Aliano	0.9838	0.385198

Bernalda	1.093028	0.01341
Calciano	0.986221	0.323357
Cirigliano	0.883794	0.193693
Colobraro	1.107808	0.241736
Craco	1.213531	0.067583
Ferrandina	1.00879	0.165211
Garaguso	1.003361	0.143251
Gorgoglion	1.063942	0.285729
Grassano	0.975795	0.256029
Grottole	1.119784	0.13005
Irsina	1.013131	0.045266
Matera	0.886804	0.001667
Miglionico	0.997743	0.24504
Montalbanc	1.113508	0.006684
Montescag	0.911186	0.001126
NovaSiri	0.994828	0.381648
OlivetoLuca	1.168132	0.171393
Pisticci	1.090716	0.004776
Policoro	1.011824	0.225637
Pomarico	1.030893	0.180935
Rotondella	1.089528	0.149008
Salandra	1.083753	0.277942
SanGiorgic	0.934573	0.18769
SanMauroF	0.96391	0.363661
Stigliano	1.003996	0.059756
Tricarico	1.040618	0.484237
Tursi	0.922667	0.002517
Valsinni	0.9769	0.340012
ScanzanoJ	1.123359	0.112439

Supplementary Table 2. SMR for cancer

Municipality	SMR (vs Ita	p-value
Abriola	1.104583	0.469651
Acerenza	0.779325	0.011052
AlbanoDiLu	0.910973	0.48204
Anzi	1.034147	0.051499
Armento	1.035524	0.111218
Atella	0.964298	0.451674
Avigliano	0.90487	0.028136
Balvano	0.815237	0.062693
Banzi	0.921554	0.389773
Baragiano	0.789321	0.01974
Barile	0.753827	0.001208
Bella	0.789289	0.001344
Brienza	0.940771	0.346088
BrindisiMor	0.882464	0.447971
Calvello	0.956105	0.168104
Calvera	0.803841	0.369541
Campomag	1.023921	0.118133
Cancellara	0.975649	0.19325
Carbone	0.674213	0.015471
SanPaoloA	1.017515	0.046636
Castelgran	0.740893	0.008506
Castellucci	0.819515	0.198242
Castellucci	1.042954	0.203227
Castelmez	0.996248	0.251952
Castelsara	0.744566	0.018332
Castronuov	0.934677	0.236094
Cersosimo	0.621306	0.00089
Chiaromon	0.961334	0.458548
CorletoPer	1.040432	0.044921
Episcopia	0.72846	0.057991
Fardella	0.989834	0.206106
Filiano	0.814914	0.019861
Forenza	0.679282	0.00091
Francavilla	0.667383	0.022628
Gallicchio	0.84861	0.00609
GenzanoDi	0.975483	0.164299
GrumentoN	0.982835	0.340989
GuardiaPe	0.772381	0.1173
Lagonegro	1.003825	0.196213
Latronico	0.840887	0.013122
Laurenzana	0.930286	0.438696
Lauria	0.775257	0.000807
Lavello	0.87915	0.003335
Maratea	0.702551	0.000741
MarsicoNu	0.764659	0.000714
Marsicovet	0.837371	0.027964
Maschito	0.810283	0.066534
Melfi	1.003921	0.389701
Missanello	1.264851	0.318046
Moliterno	1.026952	0.02568

Montemilor	1.049794	0.123462
Montemurr	0.811137	0.121295
MuroLucar	0.856448	0.015627
Nemoli	0.906753	0.48745
Noepoli	0.633059	0.001844
OppidoLuc	0.913899	0.123652
PalazzoSa	0.905852	0.09159
Pescopaga	0.989613	0.337524
Picerno	1.020354	0.001222
Pietragalla	0.75537	0.000701
Pietraperto	0.836974	0.02511
Pignola	0.923903	0.4249
Potenza	0.916824	0.225814
Rapolla	0.881916	0.00072
Rapone	1.10522	0.462438
RioneroInV	0.973016	0.294115
Ripacandid	0.892337	0.001097
Rivello	0.912223	0.273414
Roccanova	0.779429	0.00642
Rotonda	0.794935	0.05769
Ruoti	0.737632	0.000714
RuvoDelMo	0.705308	0.000668
SanChirico	0.922908	0.375925
SanChirico	0.835047	0.126265
SanCostan	0.752226	0.003957
SanFele	0.64309	0.007394
SanMartino	0.73005	0.000801
SanSeverir	0.820755	0.193756
SantAngelo	0.722297	0.141987
SantArcang	0.987634	0.389396
Sarconi	0.76581	0.05319
SassoDiCa	0.909241	0.171018
SatrianoDiI	0.686338	0.012278
SavoiaDiLu	0.697814	0.043804
Senise	0.959431	0.453057
Spinoso	0.534545	0.000706
Teana	0.825522	0.002514
TerranovaD	0.817918	0.109483
Tito	0.976146	0.177236
Tolve	0.842136	0.161048
Tramutola	0.903385	0.156983
Trecchina	0.784823	0.006177
Trivigno	0.768002	0.004083
VaglioBasil	0.899649	0.301741
Venosa	0.736315	0.086216
VietriDiPot	0.849389	0.153671
Viggianello	1.019145	0.260268
Viggiano	0.750483	0.006615
Ginestra	0.804536	0.008364
Paterno	0.914275	0.132628
Accettura	0.676438	0.00192
Aliano	0.962653	0.276969

Bernalda	0.942381	0.144116
Calciano	0.702431	0.077719
Cirigliano	0.832751	0.443403
Colobraro	1.032822	0.063192
Craco	1.037456	0.194499
Ferrandina	0.825275	0.001862
Garaguso	0.756856	0.129604
Gorgoglion	0.937322	0.469073
Grassano	0.881631	0.053571
Grottole	0.876204	0.178548
Irsina	0.759368	0.001862
Matera	0.860338	0.002125
Miglionico	0.971175	0.308316
Montalbanc	0.951135	0.318899
Montescag	0.893022	0.044258
NovaSiri	0.838127	0.008476
OlivetoLuca	0.833217	0.415508
Pisticci	0.93549	0.02883
Policoro	0.945418	0.132348
Pomarico	0.727282	0.002374
Rotondella	0.799359	0.027967
Salandra	0.955074	0.469309
SanGiorgic	0.782103	0.055727
SanMauroF	0.855823	0.175548
Stigliano	0.981402	0.263808
Tricarico	0.835324	0.004868
Tursi	0.80125	0.002097
Valsinni	0.678816	0.001913
ScanzanoJ	0.814742	0.120274

Supplementary Table 3. SHR for cardiovascular diseases

Municipality	SHR (vs Ita	p-value
Abriola	1.032298	0.379004
Acerenza	0.887997	0.073948
AlbanoDiLu	0.852013	0.067728
Anzi	0.983351	0.033387
Armento	1.925083	0.001183
Atella	0.902562	0.068342
Avigliano	0.844209	0.000745
Balvano	0.852359	0.041257
Banzi	1.649063	0.002367
Baragiano	1.120262	0.028392
Barile	1.149812	0.004175
Bella	0.725435	0.000881
Brienza	0.958336	0.48173
BrindisiMor	0.921982	0.46011
Calvello	0.943264	0.250609
Calvera	1.620672	0.001266
Campomag	0.839967	0.130778
Cancellara	0.701622	9.92E-05
Carbone	1.03957	0.308691
Castelgran	0.84283	0.104758
Castellucci	0.956959	0.391244
Castellucci	1.154982	0.12
Castelmez	0.735363	0.01226
Castelsara	1.191184	0.011265
Castronuov	1.18346	0.035481
Cersosimo	0.949548	0.30956
Chiaromon	1.333187	0.002149
CorletoPer	1.174976	0.002053
Episcopia	1.180176	0.017652
Fardella	1.010665	0.175146
Filiano	1.041781	0.355209
Forenza	1.840551	0.002219
Francavilla	1.608453	0.002255
Gallicchio	1.093629	0.3275
GenzanoDi	1.269826	0.001628
Ginestra	1.709038	0.001536
GrumentoN	1.165579	0.016689
GuardiaPe	1.672736	0.00427
Lagonegro	1.114245	0.00188
Latronico	0.76196	0.00092
Laurenzana	0.819761	0.010237
Lauria	1.188131	0.001725
Lavello	1.137929	0.001413
Maratea	1.272028	0.001425
MarsicoNu	1.080707	0.053178
Marsicovet	1.129069	0.001575
Maschito	1.786065	0.001245
Melfi	1.230634	0.001982
Missanello	0.952151	0.236731
Moliterno	0.952425	0.409848

Montemilor	1.573062	0.001655
Montemurr	2.043047	0.001655
MuroLucar	0.672078	0.000814
Nemoli	1.334521	0.001068
Noepoli	1.06291	0.470856
OppidoLuc	0.771599	0.000381
PalazzoSai	1.911806	0.001827
Paterno	1.333826	0.001601
Pescopaga	1.298657	0.001768
Picerno	0.935857	0.141447
Pietragalla	0.880235	0.013704
Pietraperto	0.823859	0.053964
Pignola	0.86393	0.000836
Potenza	0.990195	0.069158
Rapolla	1.274788	0.001641
Rapone	0.986043	0.03046
RioneroInV	1.027186	0.046244
Ripacandid	1.328238	0.001782
Rivello	1.333166	0.001562
Roccanova	1.128962	0.067539
Rotonda	1.290804	0.00131
Ruoti	1.018301	0.374769
RuvoDelMo	1.029558	0.336833
SanChirico	1.2147	0.005056
SanChirico	0.760346	0.004028
SanCostan	1.066382	0.493728
SanFele	0.943839	0.389454
SanMartinc	1.036836	0.310759
SanPaoloA	0.80068	0.183967
SanSeverir	0.983834	0.052301
SantAngelc	1.42505	0.001614
SantArcang	1.006558	0.249911
Sarconi	0.997293	0.071582
SassoDiCa	0.934139	0.434999
SatrianoDil	1.224629	0.001768
SavoiaDiLu	1.100785	0.232957
Senise	1.013606	0.467438
Spinoso	0.816564	0.018635
Teana	1.156578	0.177808
TerranovaI	0.853192	0.084949
Tito	0.733721	0.000888
Tolve	0.932616	0.24126
Tramutola	1.135636	0.006129
Trecchina	1.385616	0.001183
Trivigno	1.304579	0.006194
VaglioBasil	0.776965	0.000997
Venosa	1.45775	0.001797
VietriDiPot	1.003518	0.152152
Viggianello	1.165553	0.001461
Viggiano	1.445361	0.003243
Accettura	1.059441	0.387893
Aliano	1.17283	0.068817

Bernalda	1.179388	0.001637
Calciano	1.496851	0.002324
Cirigliano	1.243511	0.156141
Colobraro	0.926538	0.363999
Craco	1.348944	0.001743
Ferrandina	1.042962	0.163712
Garaguso	1.297082	0.001898
Gorgoglion	1.488014	0.00266
Grassano	1.174175	0.001749
Grottole	1.195995	0.002342
Irsina	1.157892	0.001571
Matera	0.947339	0.000439
Miglionico	0.900247	0.089626
Montalbanc	0.967058	0.372183
Montescag	0.838541	0.000823
NovaSiri	0.701913	0.000393
OlivetoLuca	1.363667	0.010665
Pisticci	1.204588	0.001868
Policoro	0.942002	0.021849
Pomarico	0.870617	0.006293
Rotondella	0.985414	0.168819
Salandra	1.127695	0.022381
SanGiorgic	0.935607	0.428596
SanMauroF	1.018269	0.214953
ScanzanoJ	0.940856	0.090634
Stigliano	1.415297	0.001597
Tricarico	1.479993	0.002093
Tursi	0.843462	0.000582
Valsinni	1.030865	0.32901

Supplementary Table 4. SMR for cardiovascular diseases

Municipality	SMR (vs Ita	p-value
Abriola	1.367895	0.00113
Acerenza	1.199822	0.014665
AlbanoDiLu	1.369018	0.002144
Anzi	1.143748	0.288447
Armento	1.100874	0.455008
Atella	0.903432	0.142653
Avigliano	1.253237	0.000156
Balvano	1.400201	0.000334
Banzi	1.082237	5.21E-05
Baragiano	1.169542	0.063288
Barile	0.793108	0.022098
Bella	0.970722	0.446675
Brienza	1.330011	0.000152
BrindisiMor	1.162563	0.226025
Calvello	1.378039	0.002467
Calvera	1.219813	0.166962
Campomag	1.185223	0.182104
Cancellara	1.15208	0.033512
Carbone	0.830633	0.164273
SanPaoloA	0.764426	0.057057
Castelgran	1.244687	0.018067
Castellucci	1.246331	0.06327
Castellucci	0.985925	0.144204
Castelmez	0.951747	0.46127
Castelsara	1.073536	0.497472
Castronuov	1.143731	0.310563
Cersosimo	1.32248	0.002284
Chiaromon	1.129442	0.142451
CorletoPer	1.456381	0.000408
Episcopia	1.065038	0.034991
Fardella	1.110733	0.253929
Filiano	1.384706	5.93E-05
Forenza	1.148587	0.171011
Francavilla	1.018493	0.000188
Gallicchio	0.833453	0.006542
GenzanoDi	0.806258	0.276683
GrumentoN	1.160278	0.210183
GuardiaPe	1.333431	0.038272
Lagonegro	1.332923	0.000134
Latronico	1.1659	0.012242
Laurenzana	1.152774	0.116405
Lauria	1.276947	9.7E-05
Lavello	1.037924	0.096091
Maratea	1.180324	0.017272
MarsicoNu	1.180517	0.01268
Marsicovet	1.060466	0.105002
Maschito	1.051601	0.229952
Melfi	1.085163	0.401918
Missanello	1.435924	0.014759
Moliterno	1.112531	0.207636

Montemilor	1.005002	0.171573
Montemurr	1.283174	0.021899
MuroLucar	0.945763	0.204123
Nemoli	1.408724	0.000848
Noepoli	1.54653	0.0001
OppidoLuc	1.25091	0.007761
PalazzoSai	1.214348	0.011417
Pescopaga	1.118146	0.252112
Picerno	0.966452	0.415901
Pietragalla	1.013144	0.175558
Pietraperto	1.259375	0.000259
Pignola	1.203323	0.105392
Potenza	1.09019	0.358059
Rapolla	1.039624	0.152362
Rapone	0.914263	0.181367
RioneroInV	1.136355	0.372947
Ripacandid	1.104555	0.026077
Rivello	1.014824	0.12051
Roccanova	1.2973	0.000798
Rotonda	1.13688	0.238166
Ruoti	1.431882	0.000161
RuvoDelMo	1.016587	0.055986
SanChirico	0.857584	0.16044
SanChirico	0.997771	0.152266
SanCostan	1.050176	0.272154
SanFele	1.174826	0.184261
SanMartinc	0.850733	0.006913
SanSeverir	1.013105	0.018636
SantAngelc	0.889017	0.401355
SantArcan(	1.284565	0.006987
Sarconi	1.138821	0.279304
SassoDiCa	1.148976	0.029881
SatrianoDil	1.372466	0.004498
SavoiaDiLu	1.219901	0.136711
Senise	1.356998	0.001138
Spinoso	1.059511	0.33186
Teana	1.315947	0.000105
TerranovaI	1.5275	0.000149
Tito	1.260153	0.111985
Tolve	1.245691	0.029991
Tramutola	1.125512	0.102177
Trecchina	1.151174	0.070638
Trivigno	1.147624	0.175758
VaglioBasil	1.194908	0.040486
Venosa	1.057502	0.29382
VietriDiPot	1.096285	0.372102
Viggianello	0.905412	0.007464
Viggiano	1.191743	0.035021
Ginestra	1.261153	0.001921
Paterno	1.478815	2.69E-05
Accettura	1.286978	0.001979
Aliano	0.890703	0.272809

Bernalda	1.190636	0.001647
Calciano	1.202327	0.211101
Cirigliano	0.998458	0.001177
Colobraro	1.12187	0.338148
Craco	1.622501	0.001965
Ferrandina	1.088063	0.123264
Garaguso	1.360586	0.018671
Gorgoglion	1.131599	0.417256
Grassano	1.078269	0.287559
Grottole	1.334351	0.001898
Irsina	1.207991	0.001755
Matera	0.953495	0.00168
Miglionico	1.049481	0.337369
Montalbanc	1.17381	0.005194
Montescag	0.98115	0.03755
NovaSiri	1.007583	0.199704
OlivetoLuc	1.307958	0.111543
Pisticci	1.224014	0.001341
Policoro	1.036794	0.408215
Pomarico	1.223232	0.008149
Rotondella	1.102612	0.25142
Salandra	1.200775	0.050078
SanGiorgic	1.195363	0.063039
SanMauroF	0.982742	0.134741
Stigliano	0.957303	0.445434
Tricarico	1.210641	0.001711
Tursi	1.086817	0.228512
Valsinni	1.083584	0.312047
ScanzanoJ	1.251189	0.026573

Supplementary Table 5. SMR for cardiovascular diseases, 1980-1998

Municipality	SMR (vs Ita	p-value
Abriola	0.925903	0.360772
Acerenza	1.065392	0.191435
AlbanoDiLu	1.12413	0.13429
Anzi	0.959707	0.270142
Armento	1.129741	0.150598
Atella	0.959592	0.324191
Avigliano	1.192808	0.001718
Balvano	1.089545	0.205755
Banzi	0.871612	0.099333
Baragiano	0.915757	0.232095
Barile	0.799708	0.000203
Bella	0.932283	0.184832
Brienza	1.077395	0.122561
BrindisiMor	1.33027	0.004561
Calvello	1.328859	0.008325
Calvera	1.171944	0.21337
Campomag	1.105673	0.337638
Cancellara	1.206886	0.014995
Carbone	0.994807	0.072311
SanPaoloA	1.05929	0.442558
Castelgran	1.303842	0.002067
Castellucci	1.56263	0.001878
Castellucci	1.018847	0.216523
Castelmez	0.973431	0.051155
Castelsara	0.869597	0.055071
Castronuov	1.122761	0.265434
Cersosimo	1.05398	0.345213
Chiaromon	1.196742	0.00165
CorletoPer	1.08324	0.311722
Episcopia	0.936117	0.274961
Fardella	1.173755	0.004243
Filiano	1.170501	0.002807
Forenza	1.397051	0.00285
Francavilla	0.99689	0.064151
Gallicchio	0.88476	0.004345
GenzanoDi	1.120354	0.280327
GrumentoN	1.176642	0.021313
GuardiaPe	1.471627	0.005263
Lagonegro	1.265807	0.001863
Latronico	1.262176	0.001413
Laurenzana	1.122833	0.039765
Lauria	1.158975	0.002202
Lavello	1.005873	0.379591
Maratea	1.145419	0.001833
MarsicoNu	1.035507	0.353421
Marsicovet	1.351447	0.002018
Maschito	1.229003	0.00161
Melfi	1.103359	0.002517
Missanello	1.291112	0.014239
Moliterno	1.221735	0.001878

Montemilor	1.160177	0.007404
Montemurr	1.169459	0.016513
MuroLucar	0.988371	0.001584
Nemoli	1.233841	0.007909
Noepoli	1.233254	0.006074
OppidoLuc	1.248808	0.003078
PalazzoSai	1.062531	0.106528
Pescopaga	1.048595	0.348173
Picerno	0.88716	0.075899
Pietragalla	0.992364	0.042859
Pietraperto	0.950411	0.340869
Pignola	1.082399	0.256238
Potenza	1.128647	0.045077
Rapolla	1.032866	0.010701
Rapone	0.946146	0.451129
RioneroInV	0.913471	0.333484
Ripacandid	1.01511	0.270592
Rivello	1.120679	0.05201
Roccanova	1.115186	0.034467
Rotonda	0.9137	0.225302
Ruoti	1.30412	0.001691
RuvoDelMo	1.08301	0.15195
SanChirico	0.869144	0.066964
SanChirico	0.885409	0.133329
SanCostan	1.070699	0.305604
SanFele	1.390507	0.001677
SanMartinc	1.049418	0.226021
SanSeverir	0.846097	0.07332
SantAngelc	0.882928	0.221134
SantArcang	1.057173	0.312183
Sarconi	1.075252	0.374806
SassoDiCa	1.246784	0.001344
SatrianoDil	1.249754	0.019106
SavoiaDiLu	1.213895	0.039169
Senise	1.329638	0.002255
Spinoso	0.796902	0.018809
Teana	1.097457	0.031257
TerranovaD	1.203389	0.011642
Tito	1.349655	0.009307
Tolve	1.309747	0.001746
Tramutola	1.057362	0.253415
Trecchina	1.071358	0.135256
Trivigno	1.016446	0.367489
VaglioBasil	1.093631	0.131951
Venosa	1.110324	0.247677
VietriDiPot	1.0148	0.211404
Viggianello	0.96132	0.406746
Viggiano	0.92149	0.216337
Ginestra	1.150329	0.005017
Paterno	1.07212	0.187225
Accettura	1.049882	0.376688
Aliano	1.291215	0.001405

Bernalda	1.140016	0.001251
Calciano	1.037014	0.282951
Cirigliano	0.897036	0.322587
Colobraro	1.086054	0.255522
Craco	1.328484	0.003662
Ferrandina	1.012838	0.276513
Garaguso	1.166878	0.102619
Gorgoglion	0.985268	0.00204
Grassano	1.053254	0.221183
Grottole	1.304258	0.001794
Irsina	1.130699	0.001862
Matera	0.949926	0.011812
Miglionico	1.065248	0.351023
Montalbano	1.128554	0.002911
Montescag	1.016762	0.294329
NovaSiri	0.949597	0.389154
OlivetoLuca	1.289299	0.018501
Pisticci	1.141126	0.001428
Policoro	1.012631	0.252193
Pomarico	0.9902	0.047743
Rotondella	1.019162	0.25191
Salandra	1.209565	0.002202
SanGiorgio	1.250839	0.001262
SanMauroF	1.018105	0.252797
Stigliano	1.006782	0.071822
Tricarico	1.058481	0.10008
Tursi	1.099739	0.012129
Valsinni	1.169407	0.001356
ScanzanoJ	1.082477	0.281824

Supplementary Table 6. SMR for cardiovascular diseases, 1999-2014

Municipality	SMR (vs It	p-value
Abriola	1.381987	0.000514
Acerenza	1.150745	0.043874
AlbanoDiLu	1.300648	0.005625
Anzi	1.078184	0.347936
Armento	0.991682	0.167151
Atella	0.998645	0.361049
Avigliano	1.208454	0.000448
Balvano	1.213161	0.033046
Banzi	1.014648	0.160128
Baragiano	1.180572	0.030234
Barile	0.775978	0.00387
Bella	0.983512	0.496451
Brienza	1.279668	0.000444
BrindisiMor	1.233179	0.079253
Calvello	1.403074	0.00022
Calvera	1.117929	0.428904
Campomag	1.23817	0.087413
Cancellara	1.052073	0.35118
Carbone	0.995787	0.134092
SanPaoloA	0.915883	0.37263
Castelgran	1.276868	0.003146
Castellucci	1.246406	0.047839
Castellucci	0.973029	0.197064
Castelmez	0.923885	0.251749
Castelsara	1.052263	0.286493
Castronuov	1.142899	0.257309
Cersosimo	1.316888	0.00093
Chiaromon	1.175425	0.016997
CorletoPer	1.457831	0.000416
Episcopia	1.071436	0.000219
Fardella	1.056871	0.364434
Filiano	1.318983	0.000329
Forenza	1.151158	0.09826
Francavilla	1.07016	0.386605
Gallicchio	0.838972	0.002355
GenzanoDi	0.892921	0.410233
GrumentoN	1.175911	0.122251
GuardiaPe	1.369917	0.019654
Lagonegro	1.239646	0.000426
Latronico	1.147254	0.012637
Laurenzana	1.24678	0.002964
Lauria	1.280096	0.000384
Lavello	1.045828	0.282951
Maratea	1.142272	0.036899
MarsicoNu	1.136173	0.043705
Marsicovet	1.071504	0.42693
Maschito	1.013408	0.085563
Melfi	1.059583	0.310479
Missanello	1.267372	0.088369
Moliterno	1.118755	0.118716

Montemilor	0.984619	0.383764
Montemurr	1.325082	0.00302
MuroLucar	0.947161	0.127487
Nemoli	1.424256	0.000509
Noepoli	1.37632	0.000388
OppidoLuc	1.275514	0.000649
PalazzoSa	1.198473	0.007059
Pescopaga	1.085196	0.411529
Picerno	0.924527	0.232059
Pietragalla	0.988679	0.448989
Pietraperto	1.234823	0.000487
Pignola	1.250494	0.02572
Potenza	1.085207	0.339799
Rapolla	1.016045	0.406146
Rapone	0.904691	0.070001
RioneroInV	1.112832	0.406181
Ripacandid	1.058318	0.197539
Rivello	1.090527	0.457143
Roccanova	1.347146	0.000408
Rotonda	1.078775	0.478588
Ruoti	1.351289	0.000452
RuvoDelMo	1.053231	0.391196
SanChirico	0.935999	0.29397
SanChirico	0.945281	0.414229
SanCostan	1.087461	0.178703
SanFele	1.333141	0.005075
SanMartinc	0.838554	0.002406
SanSeverir	0.898808	0.291727
SantAngelo	1.062098	0.020313
SantArcang	1.19889	0.056445
Sarconi	1.135266	0.272184
SassoDiCa	1.240969	0.000519
SatrianoDil	1.245543	0.041628
SavoiaDiLu	1.448219	0.001049
Senise	1.465668	0.000379
Spinoso	1.006984	0.078059
Teana	1.257288	0.000394
TerranovaD	1.486704	0.000441
Tito	1.233103	0.139378
Tolve	1.339132	0.000929
Tramutola	1.120848	0.085381
Trecchina	1.115706	0.140204
Trivigno	1.17475	0.056227
VaglioBasil	1.224703	0.009009
Venosa	1.078076	0.438623
VietriDiPote	1.053844	0.354393
Viggianello	0.907799	0.002479
Viggiano	1.11209	0.191002
Ginestra	1.246277	0.000935
Paterno	1.401685	0.000258
Accettura	1.213903	0.004121
Aliano	0.95066	0.396592

Bernalda	1.160091	0.001417
Calciano	1.202756	0.134794
Cirigliano	0.91719	0.45645
Colobraro	1.189693	0.055678
Craco	1.678908	0.001509
Ferrandina	1.045866	0.294658
Garaguso	1.331845	0.007631
Gorgoglion	1.251823	0.030009
Grassano	1.044978	0.415929
Grottole	1.398572	0.001162
Irsina	1.147067	0.005956
Matera	0.95225	0.00204
Miglionico	1.159719	0.057101
Montalbanc	1.142868	0.004605
Montescag	0.979012	0.148195
NovaSiri	1.014365	0.000867
OlivetoLuca	1.2688	0.104769
Pisticci	1.202688	0.001319
Policoro	1.024691	0.399921
Pomarico	1.161042	0.021994
Rotondella	1.106006	0.137667
Salandra	1.195451	0.019689
SanGiorgic	1.185998	0.030501
SanMauroF	0.968897	0.302106
Stigliano	1.005229	0.198052
Tricarico	1.183241	0.001436
Tursi	1.118375	0.022483
Valsinni	1.07706	0.223998
ScanzanoJ	1.157118	0.089569

Supplementary Table 7. SHR for respiratory diseases

Municipality	SHR (vs Ita	p-value
Abriola	1.350485	0.026064
Acerenza	0.982153	0.227765
AlbanoDiLu	0.803398	0.15844
Anzi	1.32059	0.042304
Armento	1.725736	0.002058
Atella	1.342778	0.001859
Avigliano	1.145185	0.017833
Balvano	1.114686	0.491367
Banzi	1.650108	0.002414
Baragiano	1.45672	0.001511
Barile	1.546652	0.004018
Bella	1.768517	0.001252
Brienza	1.304712	0.002055
BrindisiMor	2.00485	0.001214
Calvello	1.117175	0.421657
Calvera	1.346219	0.269735
Campomag	1.13853	0.436677
Cancellara	0.934056	0.428122
Carbone	0.596071	0.052635
Castelgran	3.081829	0.001402
Castellucci	0.554954	0.000569
Castellucci	1.032187	0.067017
Castelmez	1.094619	0.296319
Castelsara	0.782144	0.099472
Castronuov	0.905821	0.49096
Cersosimo	0.686031	0.122012
Chiaromon	0.844254	0.154772
CorletoPer	1.081566	0.416477
Episcopia	0.970297	0.249167
Fardella	0.476516	0.008016
Filiano	1.221945	0.058974
Forenza	1.769773	0.002279
Francavilla	1.186884	0.072104
Gallicchio	1.155867	0.498817
GenzanoDi	1.28248	0.001727
Ginestra	2.247485	0.00164
GrumentoN	0.906608	0.383921
GuardiaPe	2.304204	0.004101
Lagonegro	0.685743	0.000632
Latronico	0.432701	0.000828
Laurenzana	1.03638	0.124618
Lauria	0.679892	0.000533
Lavello	1.280518	0.001522
Maratea	0.734818	0.000642
MarsicoNu	1.07171	0.457106
Marsicovet	1.003962	0.077731
Maschito	1.642203	0.00136
Melfi	1.660913	0.002059
Missanello	1.702285	0.00992
Moliterno	1.031493	0.149114

Montemilor	1.369945	0.016302
Montemurr	1.908877	0.001753
MuroLucar	1.907289	0.00134
Nemoli	0.693726	0.031012
Noepoli	0.863527	0.370429
OppidoLuc	0.762999	0.004488
PalazzoSa	1.728144	0.001914
Paterno	1.437294	0.001702
Pescopaga	3.90785	0.001859
Picerno	0.984693	0.31556
Pietragalla	1.358877	0.001271
Pietraperto	1.113859	0.373359
Pignola	1.199144	0.011575
Potenza	1.136478	0.001445
Rapolla	1.904915	0.00174
Rapone	1.887173	0.002044
RioneroInV	1.70901	0.003338
Ripacandid	1.355205	0.021839
Rivello	0.550957	0.000588
Roccanova	1.216437	0.186509
Rotonda	0.919713	0.315623
Ruoti	1.188264	0.086057
RuvoDelMo	2.364375	0.002059
SanChirico	1.876031	0.001477
SanChirico	0.643686	0.006119
SanCostan	0.816315	0.316092
SanFele	1.791644	0.001792
SanMartino	1.134414	0.424119
SanPaoloA	0.82545	0.421222
SanSeverin	0.763575	0.05383
SantAngelo	2.119133	0.001714
SantArcang	1.024954	0.111942
Sarconi	0.9286	0.441742
SassoDiCa	1.099797	0.294191
SatrianoDiI	1.310617	0.018434
SavoiaDiLu	1.833524	0.001391
Senise	0.853381	0.01663
Spinoso	0.703184	0.02947
Teana	0.758762	0.238375
TerranovaI	1.13324	0.495943
Tito	0.780563	0.000802
Tolve	1.154861	0.181615
Tramutola	1.139462	0.27364
Trecchina	0.881294	0.255899
Trivigno	1.539584	0.019767
VaglioBasil	0.831883	0.146403
Venosa	1.156329	0.009185
VietriDiPote	1.075417	0.406746
Viggianello	1.125053	0.321119
Viggiano	1.500558	0.003202
Accettura	1.158761	0.193396
Aliano	0.919283	0.320235

Bernalda	1.047314	0.227597
Calciano	1.440028	0.034097
Cirigliano	1.228067	0.472596
Colobraro	0.928324	0.319539
Craco	1.539882	0.008082
Ferrandina	1.075338	0.138766
Garaguso	1.646925	0.001598
Gorgoglion	1.795739	0.002174
Grassano	1.63749	0.001483
Grottole	1.60049	0.001935
Irsina	1.182337	0.015235
Matera	0.90847	0.001464
Miglionico	1.46986	0.001407
Montalbanc	0.829078	0.017063
Montescag	0.799649	0.001116
NovaSiri	0.831109	0.022552
OlivetoLuca	2.019045	0.001708
Pisticci	0.923657	0.157455
Policoro	0.984016	0.045729
Pomarico	0.963333	0.171016
Rotondella	0.879778	0.322857
Salandra	0.946495	0.294701
SanGiorgic	1.028025	0.201178
SanMauroF	1.208307	0.11882
ScanzanoJ	1.089498	0.12355
Stigliano	1.149659	0.0532
Tricarico	2.207212	0.001746
Tursi	0.99281	0.0984
Valsinni	0.958723	0.149146

Supplementary Table 8. SMR for respiratory diseases

Municipality	SMR (vs Ita	p-value
Abriola	1.922294	0.004434
Acerenza	1.216155	0.192577
AlbanoDiLu	0.485852	0.028735
Anzi	1.019721	0.076294
Armento	1.526278	0.175606
Atella	0.886091	0.295019
Avigliano	1.223157	0.032307
Balvano	0.733333	0.278256
Banzi	0.939835	0.233772
Baragiano	0.838427	0.492451
Barile	1.061807	0.195851
Bella	0.993941	0.215711
Brienza	1.047568	0.347534
BrindisiMor	1.423971	0.230127
Calvello	1.215655	0.406982
Calvera	0.984838	0.066259
Campomag	1.166273	0.438597
Cancellara	0.691704	0.056981
Carbone	0.718764	0.332177
SanPaoloA	1.69603	0.024475
Castelgran	0.971311	0.073553
Castellucci	0.808571	0.259499
Castellucci	0.781595	0.23721
Castelmez:	0.788446	0.369204
Castelsara	0.812831	0.477708
Castronuov	0.919359	0.004571
Cersosimo	1.182437	0.433827
Chiaromon	1.185698	0.269611
CorletoPer	1.100398	0.325237
Episcopia	0.925749	0.006775
Fardella	1.011494	0.230897
Filiano	1.16715	0.425164
Forenza	0.709299	0.043536
Francavilla	0.753297	0.412443
Gallicchio	0.901901	0.383811
GenzanoDi	1.979088	0.016869
GrumentoN	1.398891	0.168849
GuardiaPe	1.591129	0.251194
Lagonegro	0.93409	0.004883
Latronico	0.930678	0.004479
Laurenzana	0.954958	0.071408
Lauria	1.250458	0.012615
Lavello	0.680175	0.000775
Maratea	0.707426	0.042725
MarsicoNu	1.124746	0.343007
Marsicovet	1.4412	0.048339
Maschito	0.778076	0.384466
Melfi	0.945612	0.182309
Missanello	0.896935	0.005306
Moliterno	0.903093	0.35794

Montemilor	1.300013	0.204513
Montemurr	1.079171	0.224944
MuroLucar	1.122675	0.29767
Nemoli	0.974041	0.105907
Noepoli	0.746284	0.329209
OppidoLuc	1.333159	0.09951
PalazzoSar	0.925203	0.160035
Pescopaga	1.063616	0.274517
Picerno	0.975451	0.070755
Pietragalla	0.989432	0.174633
Pietraperto	1.231983	0.117841
Pignola	0.629218	0.130265
Potenza	1.093833	0.494537
Rapolla	1.198663	0.004761
Rapone	0.818859	0.33472
RioneroInV	0.925618	0.005359
Ripacandid	1.011587	0.113738
Rivello	0.810864	0.444954
Roccanova	0.533541	0.004125
Rotonda	0.75764	0.370422
Ruoti	0.781746	0.254093
RuvoDelMo	0.791065	0.32988
SanChirico	1.024952	0.126212
SanChirico	1.076033	0.317499
SanCostan	0.724045	0.044769
SanFele	1.477694	0.139761
SanMartino	1.107069	0.447717
SanSeverir	1.266426	0.434017
SantAngelo	0.964481	0.006908
SantArcang	0.692094	0.120113
Sarconi	1.317435	0.251512
SassoDiCa	1.011673	0.228671
SatrianoDiI	1.399275	0.18681
SavoiaDiLu	1.03692	0.12734
Senise	1.266065	0.311442
Spinoso	0.686427	0.201003
Teana	0.750304	0.086354
TerranovaD	1.125396	0.430813
Tito	0.905498	0.004785
Tolve	0.719626	0.169081
Tramutola	1.095352	0.481747
Trecchina	0.439535	0.00031
Trivigno	0.918258	0.005372
VaglioBasil	0.425002	0.001296
Venosa	0.71048	0.334512
VietriDiPot	1.11593	0.49463
Viggianello	0.861168	0.198602
Viggiano	1.311081	0.101575
Ginestra	0.573592	0.006853
Paterno	0.927085	0.35408
Accettura	0.806458	0.423186
Aliano	0.822806	0.458913

Bernalda	0.871158	0.170368
Calciano	0.759734	0.335993
Cirigliano	1.099077	0.071916
Colobraro	0.864941	0.455122
Craco	1.237016	0.482587
Ferrandina	0.988825	0.335144
Garaguso	0.73772	0.411309
Gorgoglion	1.6247	0.06503
Grassano	1.155827	0.486908
Grottole	1.539815	0.033409
Irsina	0.7836	0.105687
Matera	0.923147	0.003441
Miglionico	0.802873	0.419391
Montalbano	1.156816	0.423075
Montescag	0.666857	0.004127
NovaSiri	0.969853	0.364782
OlivetoLuca	1.536173	0.195149
Pisticci	0.908357	0.104966
Policoro	1.058552	0.092669
Pomarico	1.068404	0.067367
Rotondella	1.286775	0.166538
Salandra	1.067883	0.037754
SanGiorgio	0.302101	0.00375
SanMauroF	1.236737	0.371509
Stigliano	1.124766	0.415146
Tricarico	0.841863	0.140093
Tursi	1.277119	0.113008
Valsinni	0.93664	0.37157
ScanzanoJ	0.749697	0.370498
